# No Evidence for Passive Gene-Environment Correlation or the Influence of Genetic Risk for Psychiatric Disorders on Adult Body Composition via the Adoption Design

**DOI:** 10.1007/s10519-020-10028-6

**Published:** 2020-11-03

**Authors:** Avina K. Hunjan, Rosa Cheesman, Jonathan R. I. Coleman, Christopher Hübel, Thalia C. Eley, Gerome Breen

**Affiliations:** 1 Social Genetic & Developmental Psychiatry Centre, Institute of Psychiatry, Psychology & Neuroscience, King’s College London, London, UK; 2 NIHR Maudsley Biomedical Research Centre, South London and Maudsley NHS Trust, London, UK; 3 Department of Medical Epidemiology and Biostatistics, Karolinska Institute, Stockholm, Sweden; 4 National Centre for Register-based Research, Department of Economics and Business Economics, Aarhus University, Aarhus, Denmark

**Keywords:** Genomics, Psychiatric disorders, Body composition, Polygenic scores, Heritability, Passive gene-environment correlation, Adoption

## Abstract

The relationship between genetic and environmental risk is complex and for many traits, estimates of genetic effects may be inflated by passive gene-environment correlation. This arises because biological offspring inherit both their genotypes and rearing environment from their parents. We tested for passive gene-environment correlation in adult body composition traits using the ‘natural experiment’ of childhood adoption, which removes passive gene-environment correlation within families. Specifically, we compared 6165 adoptees with propensity score matched non-adoptees in the UK Biobank. We also tested whether passive gene-environment correlation inflates the association between psychiatric genetic risk and body composition. We found no evidence for inflation of heritability or polygenic scores in non-adoptees compared to adoptees for a range of body composition traits. Furthermore, polygenic risk scores for anorexia nervosa, attention-deficit/hyperactivity disorder and schizophrenia did not differ in their influence on body composition traits in adoptees and non-adoptees. These findings suggest that passive gene-environment correlation does not inflate genetic effects for body composition, or the influence of psychiatric disorder genetic risk on body composition. Our design does not look at passive gene-environment correlation in childhood, and does not test for ‘pure’ environmental effects or the effects of active and evocative gene-environment correlations, where child genetics directly influences home environment. However, these findings suggest that genetic influences identified for body composition in this adult sample are direct, and not confounded by the family environment provided by biological relatives.

**Electronic supplementary material:**

The online version of this article (10.1007/s10519-020-10028-6) contains supplementary material, which is available to authorized users.

## Introduction

Body composition traits are highly heritable (Schousboe et al. 2004; Hanisch et al. 2004; Tarnoki et al. 2014). For example, in a study of 380 adult twins (230 monozygotic and 150 dizygotic pairs; male:female ratio, 68:32; age range 18–82), heritability estimates were 82% for weight, 79% for body mass index (BMI), and 74% for body fat percentage and fat-free mass, using bioelectrical impedance analysis (Tarnoki et al. 2014). Genome-wide analyses have also revealed single nucleotide polymorphism (SNP) heritability estimates of 13% for BMI (Locke et al. 2015), 10% for body fat percentage (Lu et al. 2016), 40% for fat-free mass (Medina-Gomez et al. 2017) and 10% for waist-to-hip ratio (Shungin et al. 2015). In addition, advances in polygenic scores have shown that common genetic variants account for >20% of the variance in BMI (Locke et al. 2015).

Estimates of genetic influence can be confounded by passive gene-environment correlation which refers to the association between the genotype an individual inherits from their parents and the environment in which they are raised (Kong et al. 2018). This association arises because parents not only pass genetic factors to their offspring, but also the home environment they provide. The latter is also influenced by within family genetic factors (Knafo and Jaffee 2013). Thus, both transmitted *and* non-transmitted parental genes may influence offspring by impacting how parents nurture their children (Kong et al. 2018). For example, children who inherit genetic variants associated with a higher BMI will, on average, have parents with a higher BMI with associated altered parental and family behaviours relating to food and activity levels. Therefore, the contribution of genetic factors to body composition may be overestimated because of gene-environment correlations.

Twin studies show that the home environment plays an important role in explaining the variation in BMI (Dubois et al. 2012; Schrempft et al. 2018). For example, obesity-related genes are more strongly associated with BMI in home environments characterised by poor eating and exercise habits at age 4 (Schrempft et al. 2018). However, these studies do not inform us about other body composition characteristics and were not designed to test whether parental genes contribute to the covariation between the home environment and offspring BMI. In addition, no studies have investigated whether polygenic effects on body composition are mediated through the home environment.

The adoption design provides a ‘natural experiment’ free of passive gene-environment correlation, and therefore can help elucidate whether genetic influences identified for body composition are confounded by the home environment. This is because adopted children are reared in families where they are genetically unrelated to their adoptive parents, thus genetic variance estimated for their traits result from solely direct genetic effects. In contrast, non-adopted individuals are reared by their biological parents, and therefore their traits are not only directly influenced by their own genetics, but also by passive gene-environment correlation (Plomin et al. 1985). Notably, gene-environment interaction and other forms of gene–environment correlation (evocative and active) are still present in adoptees which contribute to direct genetic effects (Plomin et al. 1977).

In UK Biobank there are a large number of individuals who were adopted in childhood (*n* = 7342). We previously explored genetic influences on educational attainment in adult adoptees and non-adoptees in the UK Biobank, and found roughly half of the predictive power of polygenic scores for educational attainment comes from passive gene–environment correlation (Cheesman et al. 2019). Similar findings have been reported for childhood educational attainment (Bates et al. 2018; Kong et al. 2018). Taken together, these findings suggested that genetic influences on educational attainment are inflated by effects of genetic variation in parents, through the home environment, and this inflation persists into adulthood. Given that childhood obesity is a strong predictor of obesity in adulthood (Simmonds et al. 2016) and that food preferences formed in childhood can carry on into adulthood (De Cosmi et al. 2017), it is plausible that important gene-environment correlations regarding body composition traits that emerge in childhood persist into adulthood.

We describe the first study to test for passive gene environment correlation on body composition traits. We also explore whether passive gene-environment correlation mediates the association between psychiatric genetic risk and body composition. This is because psychiatric disorders often present with changes in body weight and composition (Cortese et al. 2008; Fanoe et al. 2014; Lasserre et al. 2014; Manu et al. 2015; Cortese and Tessari 2017; Milaneschi et al. 2017; Bowling et al. 2018); and psychiatric symptoms are more commonly reported by individuals at the extremes of body composition (Luppino et al. 2010; Mond et al. 2011; Janney et al. 2013; Preiss et al. 2013). In addition, new research by our group has revealed that anorexia nervosa, attention-deficit/hyperactivity disorder (ADHD) and schizophrenia are genetically correlated and have significant Mendelian randomisation relations with BMI and associated body composition characteristics (Hübel et al. 2019). Thus, parental genetic variation effects on the home environment might also inflate the association between psychiatric genetic risk and body composition.

In summary, to test for passive gene-environment correlation on body composition traits, our goal was to compare the SNP-based heritability and polygenic prediction of body composition traits in a sample of adoptees and non-adoptees from the UK Biobank. Given the link between childhood and adulthood obesity, and that early food preferences can influence later food choices, we considered it likely that important passive gene-environment correlations that emerge in childhood persist into adulthood. Thus, we hypothesised that genetic effects will be increased in non-adopted individuals due to their exposure to passive gene-environment correlation in childhood. Our second goal was to test whether passive gene-environment correlation inflates associations between psychiatric genetic risk and body composition. Specifically, we hypothesised that the proportion of variance in body composition explained by polygenic risk scores (PRS) for anorexia nervosa, schizophrenia and ADHD will be increased in non-adopted individuals.

## Methods

### Study Population, Genotype Quality Control and Phenotype Definition

The UK Biobank is a large prospective cohort study consisting of approximately 500,000 participants aged 40–69 years when recruited in 2006–2010 (Sudlow et al. 2015). Participants were asked the item “Were you adopted as a child?”, to which 7342 individuals said “yes” and 492,668 individuals said “no”. Individuals that answered, “do not know” or “prefer not to answer”, or responded inconsistently, were excluded. Genome-wide genetic data came from the full release of the UK Biobank data (*N* = 487,410) and were processed according to the quality control pipeline (Bycroft et al. 2018). We restricted our analyses to individuals with full baseline phenotypic data, who also passed quality control criteria (European ancestry, unrelated; (Bycroft et al. 2018)). Our cleaned sample consisted of 6165 adopted and 370,493 non-adopted individuals. This is smaller than our previous study using this sample (Cheesman et al. 2019) due to requiring samples with complete data on more than one phenotype.

Standard genotype quality control criteria were used (Coleman et al. 2019). Genetic variants had to have a minor allele frequency > 1%, and be directly genotyped or imputed with high confidence (IMPUTE INFO metric >0.4; (McCarthy et al. 2016)). We included individuals with a genotype call rate > 98% who had concordant phenotypic and genetic gender information. The latter requiring an X-chromosome homozygosity of >0.9 and < 0.5 for phenotypic males and females, respectively. We also required individuals to be unrelated to others in the dataset using a relatedness cut-off of KING r < 0.044. This was equivalent to removing a third-degree or closer relative (Manichaikul et al. 2010). To minimise the exclusion of adoptees, we performed removal of relatives using a “greedy” algorithm (i.e. removal of the non-adoptee in a adoptee/non-adoptee duo). All analyses were restricted to individuals of European ancestry because of insufficient numbers of other ancestry groups in the adoptees (Supplementary Table 1). This was defined by 4-means clustering on the first two genetic principal components provided by the UK Biobank (Warren et al. 2017).

We examined height, BMI, body fat percentage (BF%), fat mass (FM), fat-free mass (FFM), and waist-to-hip ratio (WHR). Weight, BF%, FM and FFM were measured using the Tanita BC418MA body composition analyser (Kelly and Metcalfe 2012). This was highly standardised and used across all assessment centres. Waist and hip circumferences, and height were measured manually in the assessment centres.

### Samples

#### Propensity Score Matching

Using the R ‘MatchIt’ package (Ho et al. 2007) for propensity score analysis, we selected a matched sub-group of non-adoptees from the 370,493 non-adopted individuals in the UK Biobank. By selecting non-adopted matches for each individual adoptee, genetic differences between the groups were unlikely to reflect observed differences in phenotype or sample size. We used a ratio of 1:1 (i.e. 6165 adoptees and 6165 matched non-adoptees), matching the groups on age, sex, height, BMI, BF%, FM, FFM, WHR, weight altering medications and diagnoses that affect body composition (e.g. cancer, diabetes, Crohn’s disease, anorexia and bulimia nervosa, and major depressive disorder) (Hübel et al. 2019). Specifically, we used the nearest neighbor matching method which selects the best control match for each case one at a time (Ho et al. 2007).

**Fig. 1:**
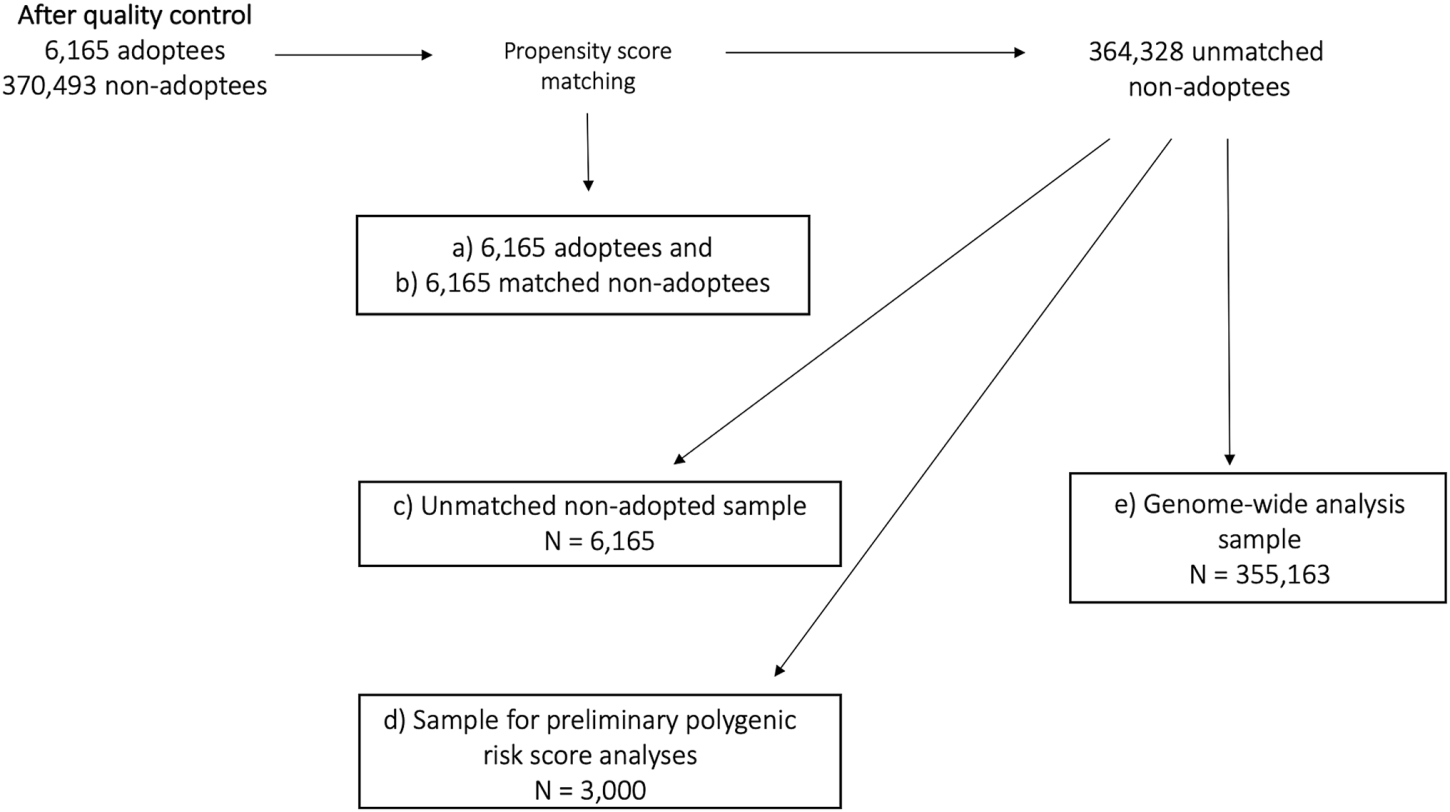
Schematic diagram summarizing the study groups

After matching, the remaining 364,328 non-adopted individuals were stratified into 3 further samples: 1) a random sample of 6165 unmatched non-adoptees to see whether adoptees and non-adoptees differ in body composition characteristics 2) a random sample of 3000 individuals to run preliminary PRS analyses to obtain optimal *p* value thresholds for each body composition trait (this sample was sufficiently powered) and 3) a large sample of 355,163 individuals to run genome-wide association studies (GWAS) to obtain SNP weights for PRS analyses. These groups have been summarised in Fig. 1.

### Phenotypic Analyses

Using the adopted and unmatched non-adopted samples (groups a and c in Fig. 1), we tested for differences in body composition between the two groups. Welsh’s Two-Sample T-tests were used to test for mean differences between adoptees and non-adoptees for parametric phenotypes (data presented as mean ± standard deviation [SD]). Wilcoxon rank sum tests with continuity correction were used to compare median differences between adoptees and non-adoptees for non-parametric phenotypes (data presented as median [interquartile range (IQR)]). To determine whether phenotypic variances differed between the two groups we used Levene’s tests. These analyses were performed in R version 3.5.3.

### Genetic Analyses

Heritability and PRS analyses of body composition traits in adoptees, matched non-adoptees and unmatched non-adoptees were controlled for age, sex, socio-economic status (SES), smoking, alcohol, menopause, pregnancy, weight altering medications, diagnoses that affect body composition, ancestral principal components 1–6, batch and centre. SES, smoking, alcohol, menopause, pregnancy, ancestral principal components, batch and centre were not included in propensity score matching because these created more heterogeneous samples, reducing the ability to closely match the groups as was possible. Beyond propensity score matching, we controlled for age, sex, weight altering medications, and diagnoses that affect body composition in adoptees and matched non-adoptees because matching does not completely control for the variable.

#### Heritability of Body Composition in Adoptees and Non-adoptees

To determine the proportion of variance explained by common variants for height, BMI, BF%, FM, FFM and WHR in adoptees, matched non-adoptees and unmatched non-adoptees, we used the genome-wide complex trait analysis (GCTA) software (v1.90b4 64-bit) to carry out Genomic-RElatedness-based restricted Maximum-Likelihood (GREML; (Yang et al. 2011)). GREML estimates heritability in unrelated individuals, avoiding the confounding of non-additive genetic and environmental effects that can occur in twin studies. The method involves creating a matrix of genomic similarity whereby genetic similarity across genotyped SNPs is calculated between each pair of individuals. This matrix is subsequently compared to a matrix of pairwise phenotypic similarity using a random-effects mixed linear model. This allows the proportion of phenotypic variance of a trait to be stratified into its genetic and residual variance component, using restricted maximum likelihood. We created three genetic relatedness matrices (adoptees, matched non-adoptees and unmatched non-adoptees) each with a relatedness cut-off of 0.044, and residualized our body composition traits via linear regression modelling. We also looked at the linkage disequilibrium (LD) score regression derived SNP-heritability of these traits in the large genome-wide analysis sample (group e in Fig. 1) (Bulik-Sullivan et al. 2015).

#### Polygenic Risk Scoring

PRSs for body composition traits were constructed via PRSice version 2.2.1 (Choi and O’Reilly 2019). SNP weights were based on the output from GWAS of each body composition trait (group e in Fig. 1).

The preliminary PRS sample of 3000 individuals (group d in Fig. 1) was used to identify the optimal *p* value threshold for inclusion of SNPs for each body composition trait. The standard set of *P*-values were tested: 0.001, 0.05, 0.1, 0.2, 0.3, 0.4, 0.5, 1. Using the optimal *p* value threshold, we ran polygenic prediction models in our adopted, matched non-adopted and unmatched non-adopted samples. To compare PRS results between the groups, we obtained bootstrapped standard errors (SE) for the R^2^ statistics using the ‘boot’ package in R, with 1000 replications. To decide whether or not to reject the null hypothesis of no difference in variance explained by PRSs in adoptees and non-adoptees, Z scores were created using the PRS R^2^ and SE.

PRSs for anorexia nervosa, schizophrenia and ADHD were also investigated due to their significant genetic overlap with body composition traits (Hübel et al. 2019). In addition, we looked at polygenic scores for educational attainment, because it has the largest Mendelian randomisation effect on body composition (Hübel et al. 2019), and childhood obesity. We used the latest summary statistics for anorexia nervosa (16,992 cases and 55,525 controls; (Watson et al. 2019)), schizophrenia (33,610 cases and 43,456 controls; (Schizophrenia Working Group of the Psychiatric Genomics Consortium 2014)), ADHD (19,099 cases and 34,194 controls; (Demontis et al. 2017)) and childhood obesity (5530 cases [≥95th percentile of BMI] and 8318 controls [<50th percentile of BMI]; (Bradfield et al. 2012)). We also used the largest GWAS summary statistics for educational attainment (*N* = 766,345; (Lee et al. 2018)). We excluded UK Biobank participants from these GWAS, and used the same procedure described above, except SNP weights were based on the output of the relevant trait GWAS.

Power calculations for our PRS analyses were performed using the ‘pwr’ package. Calculations were based on a Two Sample T-test, presuming an underlying normal distribution, a 2-sided significance level of 5% or the Bonferroni corrected threshold, and a power of 80% or greater.

#### Correction for Multiple Testing

Stringent multiple testing correction was applied to the heritability and PRS analyses, using matrix decomposition of the genetic correlation matrix of all phenotypes studied (height, BMI, BF%, FM, FFM, WHR, anorexia nervosa, schizophrenia, ADHD, educational attainment, childhood obesity), to identify the number of independent tests in order to adjust the *P* value thresholds using Bonferroni correction ((Nyholt 2004); see Supplementary materials).

## Results

### Descriptives

Detailed sociodemographic data for each group (adoptees, matched non-adoptees and unmatched non-adoptees) is provided in Table 1. Briefly, participants showed similar sociodemographic characteristics for age, sex, average total household income before tax, highest qualification, proportion of individuals with a diagnosis of anorexia nervosa, schizophrenia and ADHD, and body size at age 10.

**Table 1:** Descriptive statistics: age, sex, average total household income before tax, highest qualification, number of individuals with anorexia nervosa, schizophrenia and ADHD, and body size at age 10, separately for adoptees, matched non-adoptees and unmatched non-adoptees. Body size at age 10 was used as a proxy for childhood obesity

		Adoptees (*n* = 6165)	Matched non-adoptees (n = 6165)	Unmatched non-adoptees (n = 6165)
Age		56.37 (40–70)	56.36 (40–70)	56.81 (40–71)
Sex	Female	3226 (52.33%)	3249 (52.70%)	3339 (54.16%)
	Male	2939 (47.67%)	2916 (47.30%)	2826 (45.84%)
Average total household income before tax	Greater than 100,000	231 (3.75%)	283 (4.59%)	297 (4.82%)
	52,000 to 100,000	932 (15.12%)	1099 (17.83%)	1084 (17.58%)
	31,000 to 51,999	1295 (21.01%)	1429 (23.18%)	1456 (23.62%)
	18,000, 30,999	1356 (22%)	1298 (21.05%)	1307 (21.20%)
	Less than 18,000	1444 (23.42%)	1167 (18.93%)	1187 (19.25%)
	Do not know	257 (4.17%)	244 (3.96%)	239 (3.88%)
	Prefer not to answer	609 (9.88%)	622 (10.09%)	569 (9.23%)
	NA	41 (0.67%)	23 (0.37%)	26 (0.42%)
Highest qualification	College or university degree	1740 (28.22%)	1951 (31.65%)	2061 (33.43%)
	NVQ or HND or HNC or equivalent	424 (6.88%)	439 (7.12%)	347 (5.63%)
	Other professional qualification: e.g. nursing, teaching	337 (5.47%)	311 (5.04%)	307 (4.98%)
	A/AS levels or equivalent	727 (11.79%)	732 (11.87%)	711 (11.53%)
	O levels/GCSE or equivalent	2554 (41.43%)	2319 (37.62%)	2341 (37.97%)
	CSEs or equivalent	318 (5.16%)	349 (5.66%)	326 (5.29%)
	Prefer not to answer	60 (0.97%)	56 (0.91%)	60 (0.97%)
	NA	5 (0.08%)	8 (0.13%)	12 (0.19%)
Anorexia Nervosa		33 (0.54%)	24 (0.39%)	20 (0.33%)
Schizophrenia		30 (0.49%)	22 (0.36%)	14 (0.23%)
ADHD		6 (0.10%)	3 (0.05%)	2 (0.03%)
Body size at age 10	Thin	1996 (32.38%)	1972 (31.99%)	1981 (32.13%)
	Average	1071 (17.37%)	1097 (17.79%)	996 (16.16%)
	Plump	2932 (47.56%)	2976 (48.27%)	3075 (49.88%)
	Do not know	162 (2.63%)	120 (1.95%)	112 (1.82%)
	Prefer not to answer	4 (0.06%)	0 (0%)	1 (0.02%)

Values are mean and range (min - max) for age, otherwise values are n (%)

#### Phenotypic Analyses

The findings based on unmatched samples (groups a and c in Fig. 1) illustrate that non-adopted individuals had lower average BMI, BF%, FM, FFM and WHR than adopted individuals (Table 2). Non-adoptees also showed greater phenotypic variance than adoptees for BMI (IQR: 21.5–44 vs 21.6–41.6; *P* < 0.0001) and FM (IQR: 14.1–60.8 vs 14.8–53.7; *P* < 0.001). In contrast, non-adopted individuals showed only very slightly greater phenotypic variance for BF% (SD: 8.83 vs 8.51; P < 0.001), with significance most likely driven by the large sample sizes in this study. We compared our random sample of unmatched non-adoptees (group c in Fig. 1) to the large GWAS sample (group e in Fig. 1) and found no significant phenotypic differences (Supplementary Table 2), confirming our random sample of 6165 non-adoptees was representative of the non-adopted population in UK Biobank.

**Table 2:** Body composition characteristics of adoptees, matched non-adoptees and unmatched non-adoptees

	Adoptees (*n* = 6165)	Matched non-adoptees (n = 6165)	P	Levene’s Test	Unmatched non-adoptees (n = 6165)	P	Levene’s Test
Height	168.6 (9.27)	168.7 (9.14)	0.55^a^	ns	168.6 (9.27)	0.5^a^	ns
BMI	27.3 (21.6–41.6)	27.4 (21.5–51.3)	0.18^b^	ns	26.7 (21.5–44)	2.98 × 10^–13 b^	**
BF%	31.97 (8.83)	32.15 (8.69)	0.27^a^	ns	31.32 (8.51)	2.66 × 10^54 a^	***
FM	24.3 (14.8–53.7)	24.5 (14.8–52.9)	0.06^b^	ns	23.2 (14.1–60.8)	4.86 × 10^–8 b^	***
FFM	54.09 (11.61)	53.47 (11.56)	0.72^a^	ns	53.35 (11.5)	2.97 × 10^3 a^	ns
WHR	0.88 (0.09)	0.88 (0.09)	0.61^a^	ns	0.87 (0.09)	7.4 × 10^–11 a^	ns

Values are mean ± SD for height, body fat percentage (BF%), fat free mass (FFM) and waist-to-hip ratio (WHR)

Values are median (IQ25 - IQ75) for body mass index (BMI) and fat mass (FM)

^a^values were determined using the Welsh’s Two-Sample T-test

^b^values were determined using the Wilcoxon rank sum test with continuity correction

Significance codes for Levene’s test: 0 ‘***’ 0.001 ‘**’ 0.01 ‘*’ 0.05 ‘.’ 0.1 ns ‘1’

*N* = 12,330 for each Levene’s test; mean was used for height, BF%, FFM and WHR; median was used for BMI and FM

Following propensity score matching, the distributions for each body composition trait sufficiently overlapped, removing phenotypic differences between adopted and non-adopted individuals (Table 2). Density curve plots for each body composition trait, separately for adoptees, matched non-adoptees, unmatched non-adoptees and the full UK biobank sample, can be found in Supplementary Fig. 1.

#### Heritability and Polygenic Prediction of Body Composition in Adoptees and Non-adoptees

**Fig. 2:**
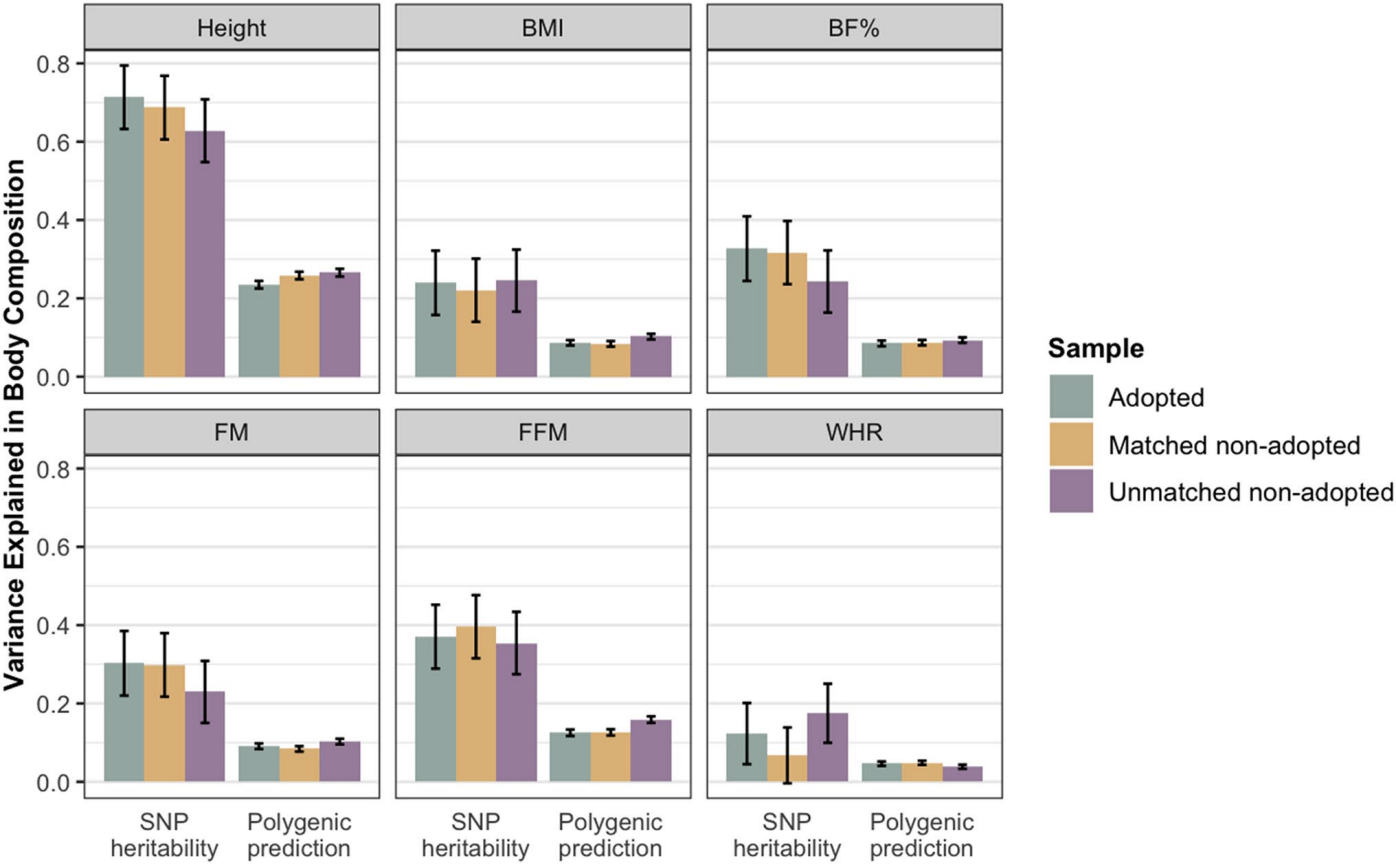
Estimates of the variance explained by common SNPs for body composition traits and by polygenic scores of each body composition trait, separately for adoptees, matched non-adoptees and unmatched non-adoptees. Error bars show standard errors. Sample sizes for polygenic-prediction analyses were 6165 for each sample; sample sizes for genomic-relatedness-based restricted-maximum-likelihood (GREML) heritability analyses were lower (6142 for adoptees, 6121 for matched non-adoptees, and 6114 for unmatched non-adoptees) due to GREML’s more strict relatedness standard. Standard errors for polygenic risk scores were obtained by bootstrapping with 1000 replications. *P* value thresholds for SNP heritability and polygenic prediction analyses after correction for multiple comparisons by matrix decomposition and Bonferroni correction were 4.16 × 10^−3^ (.05/10) and 6.94 × 10^−3^ (.05/16), respectively. None of the differences were significant. BMI = body mass index, BF% = body fat percentage, FM = fat mass, FFM = fat-free mass, WHR = waist-to-hip ratio

Figure 2 shows the variance explained by common SNPs for body composition traits and by polygenic scores of each body composition trait, separately for adoptees, matched non-adoptees and unmatched non-adoptees. We found estimates of GREML-derived SNP-based heritability for height, BMI, BF%, FM, FFM and WHR did not differ between adopted and non-adopted individuals, irrespective of matching (Fig. 2). We also observed similar results for polygenic prediction of these body composition traits in adopted and non-adopted individuals. The LD score regression derived heritability estimates for these traits are presented in Supplementary Table 3.

#### Polygenic Association of Body Composition

**Fig. 3:**
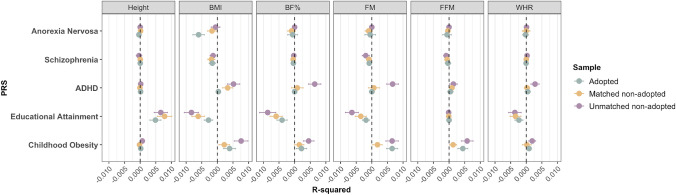
Polygenic prediction of body composition traits in adoptees, matched non-adoptees and unmatched non-adoptees, plus standard errors (bars). R-squared have been multiplied by the direction of the coefficient value. Standard errors for polygenic risk scores were obtained by bootstrapping with 1000 replications. *P* value threshold after correction for multiple comparisons by matrix decomposition and Bonferroni correction = 3.13 × 10^−3^ (.05/16). None of these findings were statistically significant. BMI = body mass index, BF% = body fat percentage, FM = fat mass, FFM = fat-free mass, WHR = waist-to-hip ratio

Figure 3 shows that the phenotypic variance in height, BMI, BF%, FM, FFM and WHR explained by PRSs for anorexia nervosa, schizophrenia, ADHD, educational attainment and childhood obesity were minimal and did not significantly differ between adopted and non-adopted individuals, irrespective of matching. We found PRSs for childhood obesity and ADHD were positively associated with BMI, BF%, FM, FFM, and WHR. In contrast, higher polygenic propensity for educational attainment was negatively associated with BMI, BF%, FM, and WHR. We found schizophrenia and anorexia nervosa PRSs showed the weakest associations across all body composition traits, the latter possibly due to low statistical power of the anorexia nervosa GWAS. Finally, PRSs for anorexia nervosa, schizophrenia, ADHD and childhood obesity were poorly associated with height, while higher polygenic propensity for educational attainment was associated with being taller. Our power calculations show that, at the sample size examined and presuming an underlying normal distribution of these body composition traits, we were sufficiently powered to detect significant differences between the groups, with an effect size of 0.05, a power of 80%, and a significance level of 0.05 (Supplementary Table 4).

## Discussion

We describe the first study to test for passive gene-environment correlations in adult body composition traits and their association with psychiatric disorders, using the adoption design. We found no significant differences in variance in body composition explained by common genetic variants or polygenic scores in adopted as compared to non-adopted individuals. We also show, for the first time, no evidence of significant differences in variance in body composition explained by PRSs for anorexia nervosa, schizophrenia and ADHD in adopted and non-adopted individuals. Our findings suggest that genetic influences on adult body composition are not magnified when individuals are reared by their close genetic relatives, with whom they share both genes and environments.

There are two potential explanations for this: i) the association between the home environment and body composition is ‘purely’ environmental i.e. does not originate in parental genotypes, or ii) parents’ genes act on offspring traits through the home environment, but these effects largely deteriorate with increasing age. Existing literature suggests that shared environmental factors that affect BMI are important in childhood but their effects largely disappear by adolescence (Silventoinen et al. 2016) and adulthood (Silventoinen et al. 2017). Similar findings were previously reported by Stunkard et al. who found the family environment has no apparent effect on being overweight in adult adoptees (Stunkard et al. 1986). These findings support the observations of our study.

A recent study revealed robust genetic correlations between childhood and adulthood BMI, with variants associated with adulthood BMI acting as early as age 4 (Couto Alves et al. 2019). However, they reported completely distinct genetics for BMI during infancy (Couto Alves et al. 2019). These findings suggest genetic influences on BMI and potentially other body composition characteristics may not be stable as differing sets of genomic variants underlie these traits in infancy versus childhood/adulthood (i.e. genetic innovation; (Silventoinen et al. 2017)). This implies that, whilst genetic variants for BMI or obesity in infancy might be affected by passive gene-environment correlation, genetic variants affecting these traits during childhood and adulthood might not be. Future research should explore passive gene-environment correlation in infancy (<4 years) and/or childhood (4–11 years).

We also observed that PRSs for anorexia nervosa, schizophrenia and ADHD did not differ in their influence on body composition traits in adoptees and non-adoptees. These findings suggest that influences of parental behaviours on offspring do not inflate the influence of psychiatric disorder genetic risk on body composition. However, whilst these PRSs were constructed from the largest available GWASs, these phenotypes still have relatively small sample sizes. Thus, it is difficult to meaningfully interpret these findings due to their potential lack of statistical power. These analyses should be repeated when sample sizes have increased.

There are several important caveats that need to be addressed when interpreting these findings. First, the UK Biobank has limited information on adoption circumstances. Data on age at adoption, adoptive parents and contact with biological parents were not collected. This information would have been valuable for the exclusion of adoptees *not* raised solely by their adoptive parents, thereby enabling a more accurate comparison between adopted and non-adopted individuals. Secondly, our design does not test for ‘pure’ environmental effects or the effects of active and evocative gene-environment correlations, where child genetics directly influences home environment. Thirdly, the UK Biobank has no information on childhood body composition, obesity or psychiatric health. Thus, we were unable to inform about passive gene environment correlation on childhood body composition traits and their associations with psychiatric disorders. We note that participants were retrospectively asked about their body size at age 10, however this is unlikely to be an accurate representation of childhood body composition. Fourthly, significant passive gene-environment correlations during prenatal development are possible. For example, maternal smoking during pregnancy (a prenatal risk factor linked to adverse changes in birth weight) is genetically-influenced, indicating the potential role of passive gene-environment correlation (Marceau et al. 2016). Future research should seek to replicate these current findings in pregnancy and birth cohorts using other designs, particularly those involving parental genotype data, to allow explicit estimation of parental effects. Finally, these analyses may be influenced by the ‘healthy and wealthy’ volunteer self-selection of European-ancestry individuals in the UK Biobank (Keyes and Westreich 2019), which makes it difficult to generalise our findings to the general population.

Nonetheless, our study has several advantages including the utilisation of a large sample size of adopted individuals for heritability and PRS analyses and a method that does not require intergenerational data. Moreover, we adjusted for multiple relevant conditions and traits. This is a unique and important feature of our investigation and substantially reduced possible confounding of our analyses. Finally, despite the caveats linked to the UK Biobank, it remains the most appropriate cohort for our investigation because no other dataset comprises sufficient phenotypic and genetic data on adopted and non-adopted individuals.

## Conclusion

The evidence presented in this study of middle-aged adults highlights that passive gene-environment correlation does not inflate overall genetic effects for, or the influence of psychiatric disorder genetic risk on, body composition. Future research should seek to replicate these findings in pregnancy and birth cohorts, and estimate the role of passive gene environment correlation on childhood body composition. If these studies also utilise parental genotype data, explicit estimates of parental effects could be calculated. Likewise, longitudinal data could help to determine if the absence of passive gene-environment correlation in adulthood results from its deterioration when offsprings’ environments are no longer influenced by their genetic relatives and if partially differing sets of genomic variants underlie these traits in childhood versus adulthood.

## Electronic supplementary material

Below is the link to the electronic supplementary material.

Supplementary file1 (DOCX 344 kb)
