# Supplementary material for: No Evidence for Passive Gene-Environment Correlation or the Influence of Genetic Risk for Psychiatric Disorders on Adult Body Composition via the Adoption Design
Source: Behav Genet. 2020 Nov 3;51(1):58–67. doi: 10.1007/s10519-020-10028-6 (PMC7815612; doi:10.1007/s10519-020-10028-6)
Supplement: Supplementary file 1 — Supplementary file1 (DOCX 344 kb) [file 10519_2020_10028_MOESM1_ESM.docx]

**Supplementary materials**

**Supplementary Table 1**: Comparison of the self-reported ethnic origins of adopted and non-adopted individuals in the UK Biobank

Excludes adopted and non-adopted participants who were missing data on ethnicity or responded “prefer not to answer” or “do not know.”

White included white British, white Irish, and other white background.

Black or black British included Caribbean, African, and other black background.

Asian or British Asian included Indian, Pakistani, Bangladeshi and other Asian background.

Mixed Included white and black Caribbean, white and black African, white and Asian, and other mixed ethnic background.

| **Ethnicity** | **Adopted (n = 7,286)** | | **Non-adopted (n = 491,101)** | |
| --- | --- | --- | --- | --- |
|  | **No. of Persons** | **%** | **No. of Persons** | **%** |
| **White** | 6,766 | 92.9 | 465,151 | 94.7 |
| **Black or black British** | 151 | 2.1 | 7,801 | 1.6 |
| **Mixed** | 163 | 2.2 | 2,763 | 0.6 |
| **Asian or Asian British** | 95 | 1.3 | 9,506 | 1.9 |
| **Chinese** | 37 | 0.5 | 1,495 | 0.3 |
| **Other ethnic group** | 74 | 1 | 4,385 | 0.9 |

**Supplementary Table 2**: Body composition characteristics of unmatched non-adoptees and remaining non-adoptees that passed quality control

Values are mean ± SD for height, body fat percentage (BF%) , fat free mass (FFM) and waist-to-hip ratio (WHR)

Values are median (Q25 - IQ75) for body mass index (BMI) and fat mass (FM)

^a^ values were determined using the Welch Two-Sample t-test

^b^ values were determined using the Wilcoxon rank sum test with continuity correction

Significance codes for Levene’s test: 0 '***' 0.001 '**' 0.01 '*' 0.05 '.' 0.1 'ns ' 1.

N=370,493 for each Levene’s test; mean was used for height, BF%, FFM and WHR; median was used for BMI and FM.

|  | **Unmatched non-adoptees** (n = 6,165) | **Non-adoptees** (n = 364,328) | **P** | **Levene’s Test** |
| --- | --- | --- | --- | --- |
| **Height** | 168.6 (9.27) | 168.77(9.24) | 0.6222^a^ | ns |
| **BMI** | 26.7 (21.5 - 44) | 27.37.7(20.6 - 47) | 0.6802^b^ | . |
| **BF%** | 31.32 (8.51) | 31.32 (8.48) | 0.9489^a^ | ns |
| **FM** | 23.2 (14.1 - 60.8) | 23.3 (14.15 - 66.6) | 0.8982^b^ | ns |
| **FFM** | 53.35 (11.5) | 53.43 (11.54) | 0.7769^a^ | ns |
| **WHR** | 0.87 (0.09) | 0.87 (0.09) | 0.7473^a^ | ns |


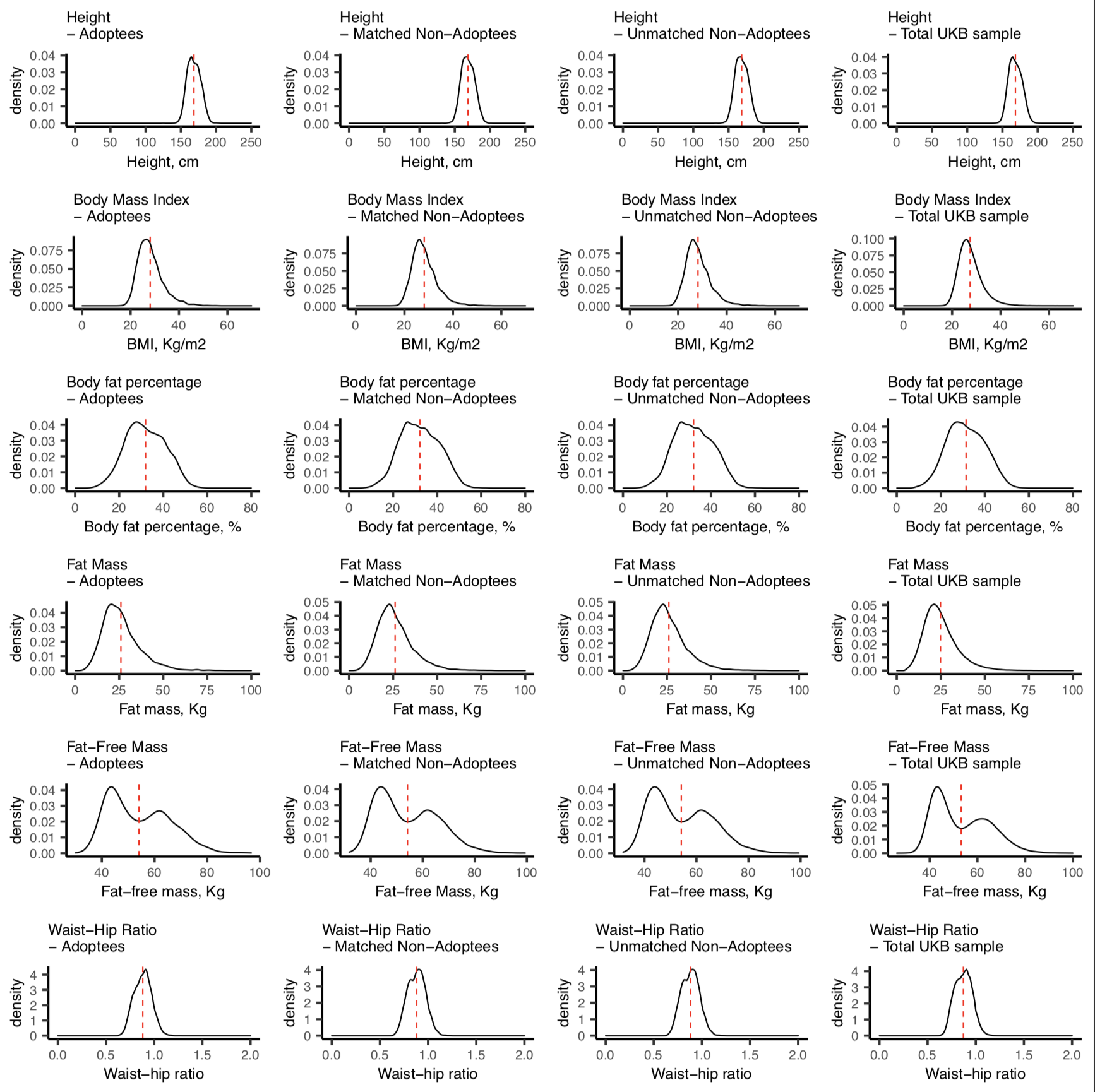
**Supplementary Figure 1**: Density curve plots for each body composition trait, separately for adoptees (column 1), matched non-adoptees (column 2), unmatched non-adoptees (column 3) and the full UK biobank sample (column 4). The red dashed line represents the mean. Sample sizes for adoptees, matched non-adoptees and unmatched non-adoptees was 6,165, and for the full UK biobank sample 491,163.

**Supplementary Table 3:** Estimates of the variance explained by common variants (SNP-based heritability derived by LDSC) for body composition traits, with standard errors.

*BMI = body mass index, BF% = body fat percentage, FM = fat mass, FFM = fat-free mass, WHR =* waist-to-hip ratio

|  | **h^2^** | **Lambda GC** | **Mean Chi^2** | **Intercept** | **Ratio** |
| --- | --- | --- | --- | --- | --- |
| 355,163 non-adopted individuals used for genome-wide association analysis (group e) | | | | | |
| **Height** | 0.424 (0.010) | 2.44 | 4.66 | 1.43 (0.03) | 0.12 (0.01) |
| **BMI** | 0.21 (0.001) | 2.01 | 2.55 | 1.08 (0.01) | 0.05 (0.01) |
| **BFP** | 0.197 (0.006) | 1.20 | 2.48 | 1.11 (0.01) | 0.07 (0.01) |
| **FM** | 0.203 (0.007) | 2.00 | 2.51 | 1.10 (0.01) | 0.07 (0.01) |
| **FFM** | 0.265 (0.01) | 2.19 | 3.16 | 1.23 (0.02) | 0.10 (0.01) |
| **WHR** | 0.14 (0.005) | 1.71 | 2.04 | 1.08 (0.01) | 0.07 (0.01) |

**GCTA-GREML and LD-score derived heritability estimates**

The GREML-derived SNP-based heritability estimates for height were large in this study (71.4% [SE=8%] in adoptees, 68.7% [SE=8%] in matched non-adoptees and 62.8% [SE = ZZ8%] in unmatched non-adoptees). However, we found the LD score regression-derived heritability estimate of height was lower 42.3% (SE = 1.9%) in the large GWAS sample of non-adopted individuals (Supplementary Table 3). This is not surprising because GCTA-GREML-derived estimates are typically higher than LD score regression estimates (Evans and Keller 2018). These differences may be the result of high LD between SNPs associated with height. This is because, in contrast to LD-score regression, no assumption is made in GREML that SNPs are in linkage equilibrium (Krishna Kumar et al. 2016). Thus, as the number of SNPs increases, the extent to which they tag the causal variant through LD may increase so that the total genetic variance explained by the SNPs increases (Krishna Kumar et al. 2016). We also found that the LD score regression-derived heritability estimates for BMI, BF%, FM, FFM and WHR in the non-adopted sample overlapped with the results found using GREML (Supplementary Table 3), suggesting low linkage equilibrium between SNPs for these body composition characteristics.

**Supplementary Table 4:** A) Raw estimates of the variance explained by common SNPs for body composition traits and by polygenic scores of each body composition trait, separately for adoptees, matched non-adoptees and unmatched non-adoptees, plus standard errors. Sample sizes for polygenic-prediction analyses were 6,165 for each sample; sample sizes for genomic-relatedness-based restricted-maximum-likelihood (GREML) heritability analyses were lower (6142 for adoptees, 6121 for matched non-adoptees, and 6114 for unmatched non-adoptees) due to GREML’s more strict relatedness standard than the KING relatedness metric. Standard errors for polygenic risk scores were obtained by bootstrapping with 1000 replications. P value thresholds for SNP heritability and polygenic prediction analyses after correction for multiple comparisons by matrix decomposition and Bonferroni correction were 4.16 x 10-3 (.05/10) and 6.94 x 10-3 (.05/16), respectively. None of the findings were significant. B) Z score calculations to obtain P values for comparisons of adoptees with matched and unmatched non-adoptees. The z scores represent the square root of the difference in estimates of explained variance (X^1^ -X^2^) divided by the differences in squared standard errors (σx^2 1^- σx^2 2^), using the values reported in A). Using these calculated Z scores, P values were then estimated using the pnorm function in R.

BMI = body mass index, BF% = body fat percentage, FM = fat mass, FFM = fat-free mass, WHR = waist-to-hip ratio.

**A**

|  | **Variance explained by SNPS** | | | **Variance explained by PRS** | | |
| --- | --- | --- | --- | --- | --- | --- |
|  | Adoptees | Matched  non-adoptees | Unmatched non-adoptees | Adoptees | Matched non-adoptees | Unmatched  non-adoptees |
| **Height** | 0.714 (0.081) | 0.687 (0.081) | 0.628 (0.080) | 0.235 (0.007) | 0.258 (0.007) | 0.266 (0.007) |
| **BMI** | 0.240 (0.082) | 0.221 (0.081) | 0.245 (0.079) | 0.086 (0.007) | 0.084 (0.007) | 0.102 (0.007) |
| **BF%** | 0.327 (0.083) | 0.317 (0.081) | 0.243 (0.079) | 0.085 (0.007) | 0.087 (0.007) | 0.093 (0.007) |
| **FM** | 0.303 (0.083) | 0.298 (0.081) | 0.229 (0.079) | 0.091 (0.007) | 0.084 (0.007) | 0.103 (0.007) |
| **FFM** | 0.371 (0.081) | 0.396 (0.081) | 0.354 (0.080) | 0.125 (0.008) | 0.126 (0.008) | 0.159 (0.008) |
| **WHR** | 0.123 (0.078) | 0.070 (0.071) | 0.175 (0.075) | 0.046 (0.006) | 0.049 (0.005) | 0.039 (0.005) |

**B**

|  | **Z score calculations - Variance explained by SNPS** | | | | | | | | | | |
| --- | --- | --- | --- | --- | --- | --- | --- | --- | --- | --- | --- |
|  | **Adoptees versus matched non-adoptees** | | | | | **Adoptees versus unmatched non-adoptees** | | | | | |
|  | X^1^ -X^2^ | σx^2 1^- σx^2 2^ | Square root of σx^2 1^- σx^2 2^ | Z | P | X^1^ -X^2^ | σx^2 1^- σx^2 2^ | Square root of σx^2 1^- σx^2 2^ | Z | | P |
| **Height** | 0.027 | 0.013 | 0.115 | 0.231 | 0.817 | 0.085 | 0.013 | 0.114 | 0.750 | | 0.453 |
| **BMI** | 0.019 | 0.013 | 0.115 | 0.165 | 0.869 | -0.006 | 0.013 | 0.114 | -0.049 | | 0.961 |
| **BF%** | 0.010 | 0.013 | 0.115 | 0.087 | 0.930 | 0.084 | 0.013 | 0.115 | 0.733 | | 0.464 |
| **FM** | 0.004 | 0.013 | 0.116 | 0.036 | 0.971 | 0.073 | 0.013 | 0.114 | 0.640 | | 0.522 |
| **FFM** | -0.025 | 0.013 | 0.115 | -0.222 | 0.824 | 0.016 | 0.013 | 0.114 | 0.142 | | 0.887 |
| **WHR** | 0.056 | 0.011 | 0.106 | 0.527 | 0.599 | -0.052 | 0.012 | 0.109 | -0.478 | | 0.632 |
|  | **Z score calculations - Variance explained by PRS** | | | | | | | | | | |
|  | **Adoptees versus matched non-adoptees** | | | | | **Adoptees versus unmatched non-adoptees** | | | | | |
|  | X^1^ -X^2^ | σx^2 1^- σx^2 2^ | Square root of σx^2 1^- σx^2 2^ | Z | P | X^1^ -X^2^ | σx^2 1^- σx^2 2^ | Square root of σx^2 1^- σx^2 2^ | Z | P | |
| **Height** | -0.024 | 0.000 | 0.010 | -2.394 | 0.017 | 0.031 | 0.000 | 0.010 | 3.118 | 0.002 | |
| **BMI** | 0.003 | 0.000 | 0.010 | 0.267 | 0.790 | 0.016 | 0.000 | 0.010 | 1.607 | 0.108 | |
| **BF%** | -0.002 | 0.000 | 0.010 | -0.188 | 0.851 | 0.008 | 0.000 | 0.010 | 0.837 | 0.403 | |
| **FM** | 0.007 | 0.000 | 0.010 | 0.679 | 0.497 | 0.012 | 0.000 | 0.010 | 1.186 | 0.236 | |
| **FFM** | -0.001 | 0.000 | 0.011 | -0.082 | 0.935 | 0.034 | 0.000 | 0.012 | 2.869 | 0.004 | |
| **WHR** | -0.002 | 0.000 | 0.008 | -0.290 | 0.772 | -0.008 | 0.000 | 0.008 | -1.036 | 0.300 | |

**Supplementary Table 5:** A) Raw estimates of the variance explained by polygenic scores for anorexia nervosa, schizophrenia, ADHD, educational attainment and childhood obesity, separately for adoptees, matched non-adoptees and unmatched non-adoptees, plus standard errors. Estimates have been multiplied by 100 to percentages. Sample sizes for polygenic-prediction analyses were 6,165 for each sample. Standard errors for polygenic risk scores were obtained by bootstrapping with 1000 replications. The P value threshold for polygenic prediction analyses after correction for multiple comparisons by matrix decomposition, which estimates the true number of independent tests, and subsequent Bonferroni correction was 6.94 x 10-3 (.05/16). None of the findings were significant after correcting for multiple comparison. B) Z score calculations to obtain P values for comparisons of adoptees with matched and unmatched non-adoptees. The z scores represent the square root of the difference in estimates of explained variance (X^1^ -X^2^) divided by the differences in squared standard errors (σx^2 1^- σx^2 2^), using the values reported in A). Using these calculated Z scores, P values were then estimated using the pnorm function in R.

BMI = body mass index, BF% = body fat percentage, FM = fat mass, FFM = fat-free mass, WHR = waist-to-hip ratio.

**A**

|  | **Anorexia Nervosa** | | | **Schizophrenia** | | |
| --- | --- | --- | --- | --- | --- | --- |
|  | Adoptees | Matched non-adoptees | Unmatched non-adoptees | Adoptees | Matched non-adoptees | Unmatched non-adoptees |
| **Height** | -0.04 (0.054) | 0.014 (0.07) | 0 (0.044) | 0.001 (0.03) | 0.002 (0.034) | -0.036 (0.089) |
| **BMI** | -0.602 (0.185) | -0.173 (0.141) | -0.059 (0.141) | -0.162 (0.101) | -0.193 (0.115) | -0.138 (0.096) |
| **BF%** | -0.063 (0.159) | -0.097 (0.122) | 0 (0.034) | -0.039 (0.055) | -0.045 (0.06) | -0.027 (0.044) |
| **FM** | -0.052 (0.172) | -0.101 (0.113) | 0 (0.037) | -0.082 (0.075) | -0.093 (0.081) | -0.194 (0.114) |
| **FFM** | -0.064 (0.139) | -0.038 (0.063) | 0 (0.05) | -0.035 (0.088) | -0.062 (0.064) | -0.092 (0.082) |
| **WHR** | -0.022 (0.091) | -0.002 (0.091) | 0.009 (0.121) | -0.005 (0.031) | 0.001 (0.031) | 0.026 (0.059) |
|  | **ADHD** | | | **Educational Attainment** | | |
|  | Adoptees | Matched non-adoptees | Unmatched non-adoptees | Adoptees | Matched non-adoptees | Unmatched non-adoptees |
| **Height** | 0.014 (0.054) | -0.008 (0.033) | 0.018 (0.038) | 0.489 (0.177) | 0.781 (0.231) | 0.66 (0.213) |
| **BMI** | 0.032 (0.073) | 0.329 (0.161) | 0.517 (0.2) | -0.284 (0.138) | -0.615 (0.204) | -0.831 (0.229) |
| **BF%** | 0 (0.075) | 0.087 (0.186) | 0.644 (0.198) | -0.401 (0.163) | -0.592 (0.198) | -0.861 (0.259) |
| **FM** | 0.001 (0.082) | 0.089 (0.087) | 0.664 (0.188) | -0.187 (0.112) | -0.354 (0.18) | -0.637 (0.21) |
| **FFM** | 0.044 (0.087) | 0.089 (0.087) | 0.139 (0.122) | 0 (0.07) | -0.01 (0.048) | -0.013 (0.042) |
| **WHR** | 0.047 (0.085) | 0.025 (0.084) | 0.281 (0.131) | -0.234 (0.129) | -0.344 (0.178) | -0.364 (0.201) |
|  | **Childhood obesity** | | |  |  |  |
|  | Adoptees | Matched non-adoptees | Unmatched non-adoptees |  |  |  |
| **Height** | 0.017 (0.039) | 0.039 (-0.02) | 0.065 (0.066) |  |  |  |
| **BMI** | 0.395 (0.191) | 0.191 (0.216) | 0.758 (0.222) |  |  |  |
| **BF%** | 0.224 (0.167) | 0.167 (0.146) | 0.449 (0.17) |  |  |  |
| FM | 0.647 (0.183) | 0.183 (0.126) | 0.647 (0.203) |  |  |  |
| FFM | 0.437 (0.162) | 0.162 (0.126) | 0.584 (0.188) |  |  |  |
| WHR | 0.087 (0.075) | 0.024 (0.135) | 0.758 (0.222) |  |  |  |

**B**

|  | **Adoptees versus matched non-adoptees** | | | | | **Adoptees versus unmatched non-adoptees** | | | |  |
| --- | --- | --- | --- | --- | --- | --- | --- | --- | --- | --- |
|  | X^1^ -X^2^ | σx^2 1^- σx^2 2^ | Square root of σx^2 1^- σx^2 2^ | Z | P | X^1^ -X^2^ | σx^2 1^- σx^2 2^ | Square root of σx^2 1^- σx^2 2^ | Z | P |
|  | **Anorexia Nervosa** | | | | | | | | | |
| **Height** | -0.054 | 0.008 | 0.088 | -0.609 | 0.543 | 0.040 | 0.005 | 0.070 | 0.575 | 0.565 |
| **BMI** | -0.429 | 0.054 | 0.232 | -1.848 | 0.065 | 0.543 | 0.054 | 0.232 | 2.340 | 0.019 |
| **BF%** | 0.034 | 0.040 | 0.201 | 0.171 | 0.864 | 0.062 | 0.026 | 0.163 | 0.384 | 0.701 |
| **FM** | 0.049 | 0.043 | 0.206 | 0.238 | 0.812 | 0.052 | 0.031 | 0.176 | 0.293 | 0.769 |
| **FFM** | -0.026 | 0.023 | 0.153 | -0.170 | 0.865 | 0.064 | 0.022 | 0.148 | 0.432 | 0.666 |
| **WHR** | -0.020 | 0.016 | 0.128 | -0.158 | 0.875 | 0.031 | 0.023 | 0.152 | 0.206 | 0.837 |
|  | **Schizophrenia** | | | | | | | | | |
| **Height** | -0.001 | 0.002 | 0.046 | -0.027 | 0.979 | -0.037 | 0.009 | 0.094 | -0.396 | 0.692 |
| **BMI** | 0.031 | 0.023 | 0.153 | 0.200 | 0.841 | 0.024 | 0.019 | 0.140 | 0.172 | 0.863 |
| **BF%** | 0.005 | 0.007 | 0.081 | 0.067 | 0.947 | 0.012 | 0.005 | 0.070 | 0.172 | 0.863 |
| **FM** | 0.011 | 0.012 | 0.110 | 0.097 | 0.923 | -0.112 | 0.019 | 0.136 | -0.824 | 0.41 |
| **FFM** | 0.027 | 0.012 | 0.109 | 0.247 | 0.805 | -0.057 | 0.015 | 0.121 | -0.471 | 0.638 |
| **WHR** | -0.006 | 0.002 | 0.044 | -0.144 | 0.885 | 0.032 | 0.004 | 0.066 | 0.479 | 0.632 |
|  | **ADHD** | | | | | | | | | |
| **Height** | 0.022 | 0.004 | 0.063 | 0.347 | 0.728 | 0.004 | 0.004 | 0.066 | 0.063 | 0.949 |
| **BMI** | -0.297 | 0.031 | 0.177 | -1.682 | 0.093 | 0.485 | 0.045 | 0.213 | 2.277 | 0.023 |
| **BF%** | -0.087 | 0.040 | 0.200 | -0.435 | 0.664 | 0.644 | 0.045 | 0.212 | 3.035 | 0.002 |
| **FM** | -0.088 | 0.014 | 0.120 | -0.733 | 0.464 | 0.663 | 0.042 | 0.205 | 3.230 | 0.001 |
| **FFM** | -0.045 | 0.015 | 0.123 | -0.363 | 0.716 | 0.095 | 0.022 | 0.150 | 0.635 | 0.525 |
| **WHR** | 0.022 | 0.014 | 0.120 | 0.185 | 0.853 | 0.234 | 0.024 | 0.156 | 1.499 | 0.134 |
|  | **Educational attainment** | | | | | | | | | |
| **Height** | -0.292 | 0.085 | 0.291 | -1.003 | 0.316 | 0.171 | 0.077 | 0.277 | 0.616 | 0.538 |
| **BMI** | 0.330 | 0.061 | 0.246 | 1.341 | 0.18 | -0.547 | 0.071 | 0.267 | -2.045 | 0.041 |
| **BF%** | 0.191 | 0.066 | 0.257 | 0.744 | 0.457 | -0.461 | 0.094 | 0.307 | -1.502 | 0.133 |
| **FM** | 0.167 | 0.045 | 0.212 | 0.787 | 0.431 | -0.450 | 0.057 | 0.238 | -1.890 | 0.059 |
| **FFM** | 0.010 | 0.007 | 0.085 | 0.115 | 0.908 | -0.013 | 0.007 | 0.081 | -0.165 | 0.869 |
| **WHR** | 0.110 | 0.048 | 0.220 | 0.503 | 0.615 | -0.130 | 0.057 | 0.239 | -0.542 | 0.588 |
|  | **Childhood obesity** | | | | | | | | | |
| **Height** | 0.037 | 0.003 | 0.058 | 0.643 | 0.52 | 0.048 | 0.006 | 0.077 | 0.621 | 0.535 |
| **BMI** | 0.179 | 0.065 | 0.255 | 0.702 | 0.482 | 0.363 | 0.086 | 0.293 | 1.239 | 0.215 |
| **BF%** | 0.078 | 0.052 | 0.227 | 0.345 | 0.73 | 0.224 | 0.057 | 0.238 | 0.943 | 0.186 |
| **FM** | 0.521 | 0.047 | 0.216 | 1.933 | 0.053 | 0.000 | 0.075 | 0.273 | 0.000 | 0.07 |
| **FFM** | 0.311 | 0.039 | 0.198 | 1.569 | 0.117 | 0.148 | 0.061 | 0.248 | 0.596 | 0.037 |
| **WHR** | 0.063 | 0.024 | 0.155 | 0.409 | 0.683 | 0.671 | 0.055 | 0.234 | 2.862 | 0.005 |

**Supplementary Table 6:** Power calculations for PRS analyses. Sample size is number in *each* group.

| **Effect size** | **Sample Size** | **Significance level** | **Power** |
| --- | --- | --- | --- |
| 0.05 | 6165 | 0.05 | 0.8 |
|  | 8407 | 0.05 | 0.9 |
|  | 11532 | 3.13 x 10^-3^ | 0.8 |
| 0.1 | 1571 | 0.05 | 0.8 |
|  | 2102 | 0.05 | 0.9 |
|  | 2885 | 3.13 x 10^-3^ | 0.8 |

**Matrix decomposition to identify number of independent tests**

We used a Bonferroni threshold (by correcting α as α/N) to estimate the number of significant genetic correlations, with N = estimated number of independent tests. We built a similarity matrix reflecting the trait similarity where D = number of principal components accounting for 99.5% of the data variance in the genetic correlation matrix. In that case, D is the estimated number of independent traits (GWAS), and the number of independent tests can be computed as N = (D(D-1))/2.
